# Radiographic Findings of an Intracranial Germinoma in a 42-Year-Old Male

**DOI:** 10.7759/cureus.27535

**Published:** 2022-07-31

**Authors:** Iger Ostreni, Menachem Gurevitz, Gerard Morvillo

**Affiliations:** 1 Medicine, Touro College of Osteopathic Medicine, New York City, USA; 2 Medicine, Mercy Catholic Medical Center, Philadelphia, USA; 3 Pathology, Northwell Health Staten Island University Hospital, New York City, USA

**Keywords:** adult, incontinence, weakness, radiation, cd 117, c-kit, magnetic resonance imaging, computed tomography, pineal germinoma

## Abstract

Germ cell tumors are rare tumors that most commonly occur in the pediatric population. Their usual location is in the pineal gland and above the suprasellar cistern. Pure germinomas, the most common type of germ cell tumor, are exquisitely sensitive to radiation, and rarely recur after radiotherapy treatment. We present a case of a pure germinoma that (1) occurred in the cerebellar hemisphere, (2) in the fifth decade of life, and (3) after being adequately treated with radiotherapy.

## Introduction

Intracranial germ cell tumors are a heterogeneous group of tumors that commonly arise in the pineal gland and the suprasellar region. They are especially common in the pediatric population, where they account for about 3% of all pediatric tumors in Western countries, and up to 11% of pediatric tumors in Japan [1-2]. Racial, familial, and unknown environmental factors may play a role in the high incidence of tumor development observed in that region [3]. According to the World Health Organization, there are several types of germ cell tumors, including embryonal carcinoma, yolk sac tumor, choriocarcinoma, benign teratomas, immature teratomas, mature teratomas with malignant transformation, and mixed germ cell tumors. Pure germinomas account for 50-70% of all germ cell tumors [4]. Despite the various types of germ cell tumors that exist, accurate diagnosis is essential because treatment modalities and prognosis differ significantly among the various types of tumors.

## Case presentation

A 42-year-old male presented to the emergency department due to a three-week history of change in behavior. His wife reported that he had difficulty understanding and processing information, frequently slurred his words, and his text messages contained an unusual amount of spelling errors. He also had difficulty standing from a lying position, lower extremity weakness, and fecal incontinence, although he usually remained unaware of the incontinence until others told him about it.

His medical conditions included hereditary hepatitis B and cirrhosis, and a pineal germinoma 17 years ago. The germinoma was effectively treated with radiotherapy, and a ventriculoperitoneal shunt was placed for symptomatic relief. The ventriculoperitoneal shunt was removed once it was no longer indicated. Additionally, he was treated for esophageal varices three times in the past, and was removed from the liver transplant list due to end-stage liver disease. 

**Figure 1:**
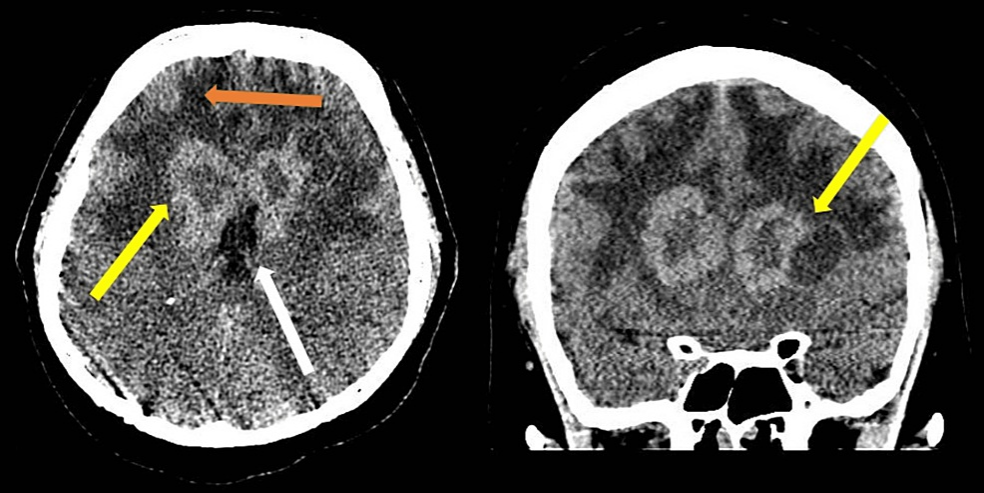
CT images from the base of skull to vertex without IV contrast. There is bilateral frontal lobe periventricular hyperdense lesions with areas of cystic changes (yellow arrows) and extensive surrounding cerebral edema (orange arrow). There is effacement of bilateral lateral ventricles (white arrow).

As part of his initial workup, a computed tomography (CT) of his head was performed, which showed bilateral frontal lobe periventricular hyperdense lesions with areas of cystic change and extensive surrounding cerebral edema (Figure 1). 

**Figure 2:**
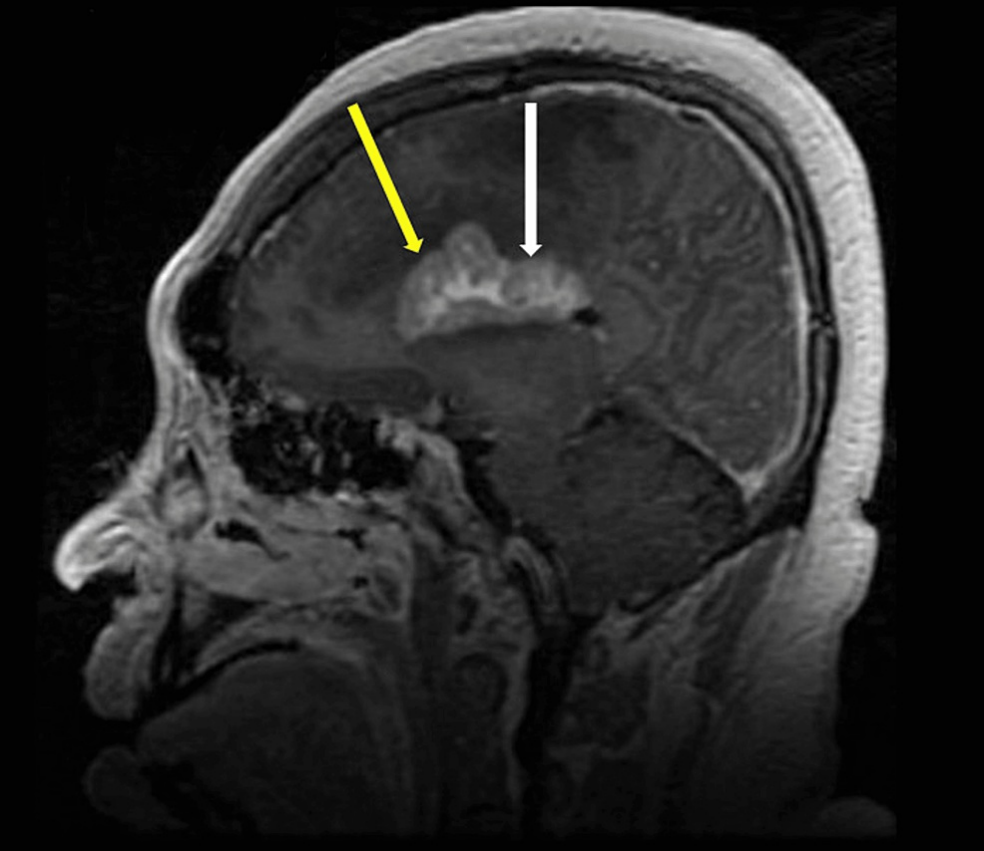
Sagittal T1 Post-Contrast. There are large bilateral heterogeneously enhancing masses centered within the body (white arrow) and genu (yellow arrow) of the corpus callosum.

**Figure 3:**
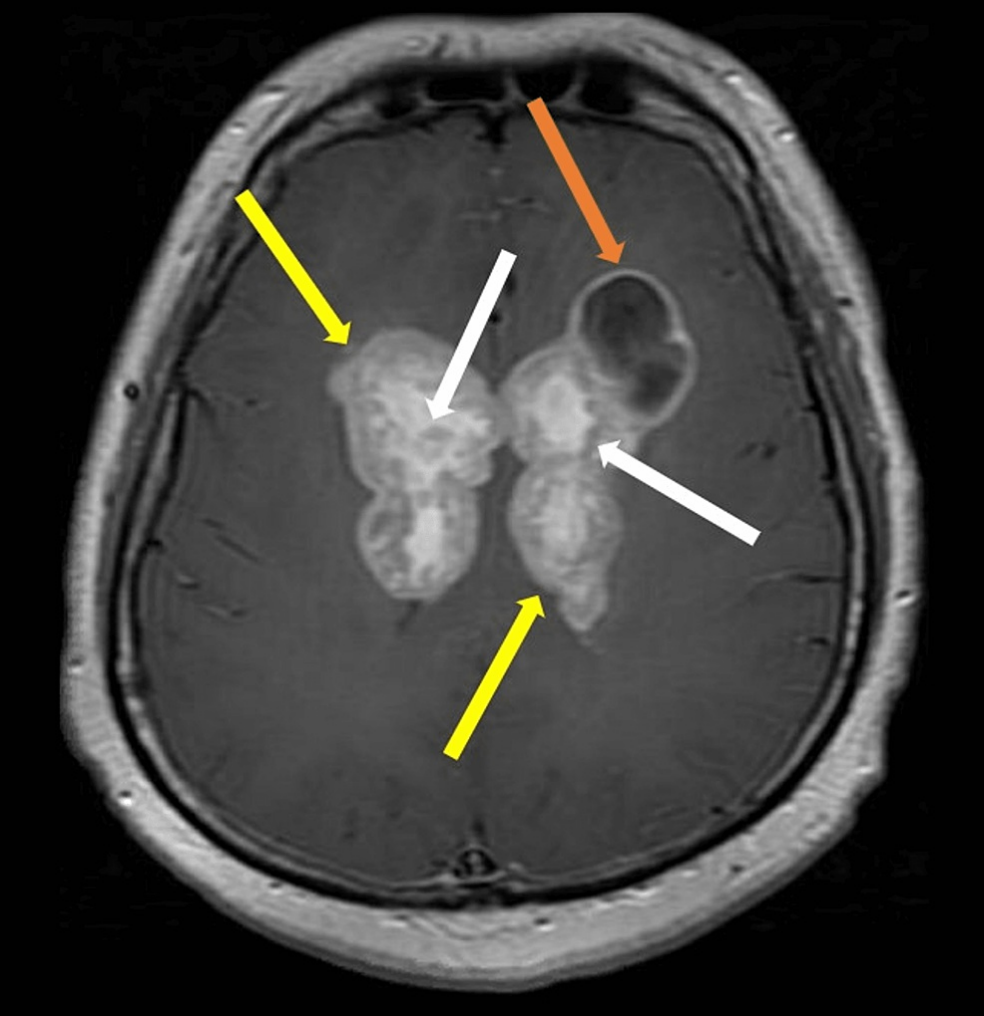
Axial Post-Contrast T1. There is extension laterally into the parasagittal deep frontal and parietal parasagittal (yellow arrows) white matter. There is associated internal cystic change (white arrows) within the mass as well as mild mass effect upon the lateral ventricles. There is a dominant peripherally enhancing tumoral cyst involving the anterior aspect of the left parasagittal tumor (orange arrow). There is no extra-axial fluid collection.

**Figure 4:**
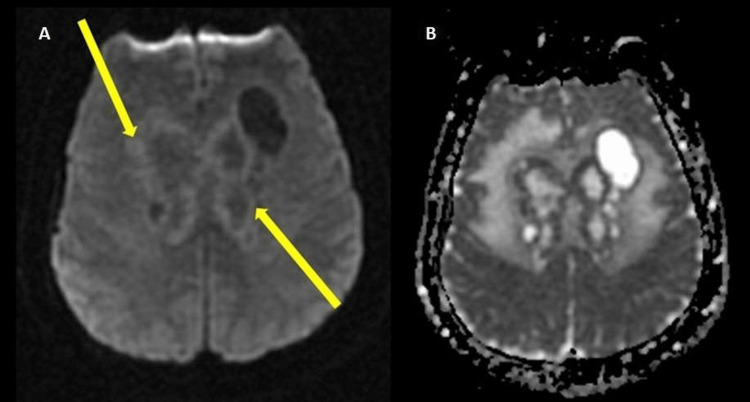
DIffusion-weighted imaging (A) and apparent diffusion coefficient map (B). The periphery of the lesions display slightly increased signal on diffusion-weighted imaging (yellow arrows).

**Figure 5:**
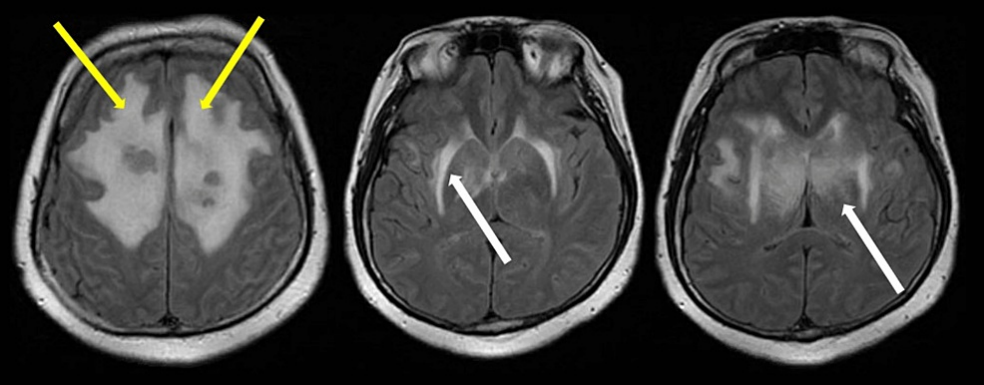
T2 FLAIR images. There is significant vasogenic edema involving the bilateral frontal lobes (yellow arrows) as well as the basal ganglia (white arrows). FLAIR: fluid attenuated inversion recovery

The lesions were further characterized by contrast-enhanced MRI of the brain which revealed large infiltrative enhancing masses involving the corpus callosum (Figure 2) and bilateral parasagittal frontal and parietal deep white matter (Figure 3) likely reflecting a high-grade glioma or lymphoma. On diffusion-weighted imaging, the periphery of the lesion demonstrated slightly increased diffusion restriction (Figure 4). Further characterization on T2 fluid-attenuated inversion recovery (FLAIR) showed areas of significant vasogenic edema in bilateral frontal lobes and basal ganglia (Figure 5).

Because of our patient’s age and the tumor’s bilateral location in the cerebral hemisphere, the initial differential included glioblastoma and primary CNS lymphoma. Despite a history of germinoma, it was given a low probability in the initial differential diagnosis since it was more than 17 years prior to the current presentation and because of its location outside the pineal gland and the suprasellar region. 

**Figure 6:**
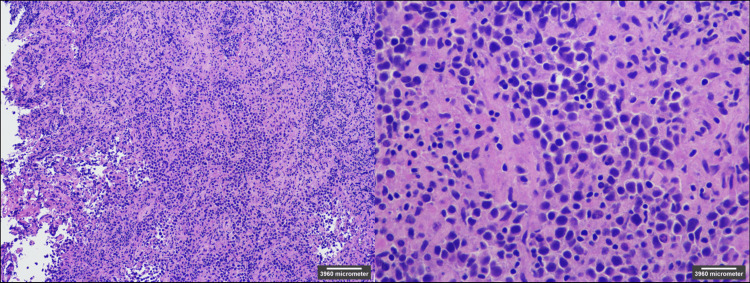
H&E stain, 10x (left) and H&E stain, 40x (right). Stained sections show atypical large tumor cells with associated prominent lymphocytic reactive cells indicative of a pure germinoma. H&E: hematoxylin and eosin

**Figure 7:**
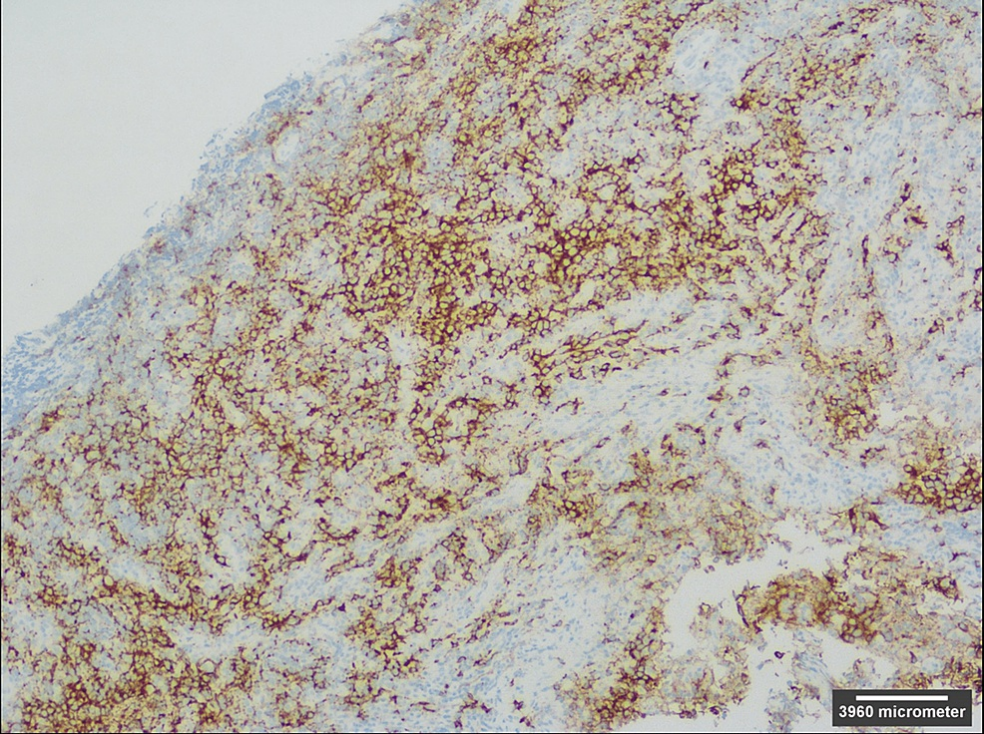
Positive immunohistochemical staining for c-kit (CD 117, receptor tyrosine kinase protein) 10x.

A brain biopsy was subsequently performed and a follow-up CT of the head without contrast showed stable intra-parenchymal hemorrhage along the biopsy tract (not shown). On histologic examination of the sample, a germinoma was observed (Figure 6) that stained positive for both c-kit (Figure 7), a marker for germinoma [5], and D2-40, a marker for seminoma (not shown) [6]. Serum alpha-fetoprotein (AFP) and beta-human chorionic gonadotropin (HCG) were negative, making the mass most consistent with a pure germinoma.

## Discussion

Although the origin and etiology of germinomas remain unclear, two prevailing theories exist. One theory is that primordial germ cells seed along the midline axis as they migrate to their gonads during embryological development. The second theory is that germs cells play an unknown regulatory role in various organs and locations, such as the thymus, the retroperitoneum, and the brain [7]. Once present in the aforementioned locations, unknown genetic or environmental conditions enable germ cells to grow and metastasize.

Pure germinomas are the most common germ cell tumors and are distinguished from other germ cell tumors by the lack of alpha-fetoprotein and minimal (<50 mcg/L) to no beta HCG. Symptoms vary based on the location, and the associated compression of surrounding structures. Common presenting features include endocrine abnormalities, nausea, vomiting, dizziness, and signs of increased intracranial pressure.

Germinomas are exquisitely sensitive to radiation therapy, with a five-year survival rate greater than 90%. However, because of the long-term effects of radiation, a combination of chemotherapy and limited radiation has been adapted with a comparable success rate [1,2,8]. Recurrence, however, is not uncommon, and protracted follow-up is advised [9]. In these instances, because the individual was already subjected to radiation, a combination of chemotherapy and radiation is strongly advised, as opposed to radiation therapy alone [10,11]. Furthermore, it has been suggested that even at its initial presentation, radiation should be limited to “reduced-volume-radiotherapy plus boost” instead of craniosacral radiotherapy [12].

Several factors make our case unique. First, germ cell tumors most commonly affect children and adolescents, whereas our patient was in his fifth decade of life. Second, less than 15% of germinomas reoccur; when they reoccur, the time frame is not as protracted as in our case. Hu et al. identified 88 patients from the literature with a recurrent intracranial germinoma, with the median being 30.3 months and a range of 3.3-134.9 months [9]. By contrast, our patient’s recurrence was after 204 months; 69 months after the most protracted case in his range. Third, the most common site for intracranial germinomas is the pineal gland or suprasellar region, whereas our patients’ germinomas presented in the corpus callosum and cerebral hemisphere. Although, keeping in mind that our patient had a distant history of germinoma, we certainly appreciate the possibility that our case was an incident of a secondary germinoma that lay dormant in the pineal gland and later metastasized. Fourth, when germinomas do reoccur, it is usually when treatment is limited to chemotherapy, or chemotherapy plus a minimal dose of radiation. Our patient, however, received a maximum dose of radiation upon his initial diagnosis. This last point presented a unique challenge in his treatment as radiotherapy remains the best curative option and is far superior to chemotherapy alone. Future treatment options can explore treatment with a combination of full dose radiation therapy and chemotherapy to minimize recurrence rates.

## Conclusions

While germ cell tumors are often thought of as pediatric tumors affecting the pineal gland or suprasellar cistern, exceptions do occur. Unlike glioblastoma or other tumors affecting the cerebellar hemisphere, pure germinomas are exquisitely sensitive to radiation therapy, making an accurate diagnosis essential to patient care. A conclusive diagnosis can be established through histological inspection and immunohistochemical stains; however, in the event that a biopsy is not possible, tumor markers and clinical history should be utilized to aid in the diagnosis. Although new chemotherapy modalities are currently under investigation, radiation therapy still remains the mainstay of treatment. 
